# Psychological and neurological benefits of high-quality nursing for postoperative hypertensive intracranial hemorrhage patients

**DOI:** 10.3389/fneur.2025.1627446

**Published:** 2025-09-24

**Authors:** Lili Feng, Lanli Zhu, Bing Wang

**Affiliations:** Department of Neurosurgery, Lixin County Hospital of Traditional Chinese Medicine, Bozhou, China

**Keywords:** hypertensive, intracranial hemorrhage, high-quality nursing, psychological state, neurological function, daily living ability

## Abstract

**Objectives:**

High-quality nursing, defined as evidence-based, patient-centered care that integrates standardized physical care with individualized psychological support, epitomizes superior nursing service delivery. The present study aimed to investigate the effects of High-quality nursing intervention on neurological function, psychological state, and quality of life in postoperative patients with hypertensive intracranial hemorrhage (ICH).

**Methods:**

This study enrolled patients who had undergone surgery for ICH between August 2019 and January 2025. Patients were randomly assigned into two cohorts: an intervention group and a control group. The control group received standard conventional nursing care, while the intervention group underwent high-quality nursing interventions. Comparative analyses were conducted between the two groups regarding various parameters, including neurological function, assessed via the National Institutes of Health Stroke Scale (NIHSS) and the Barthel Index scores; psychological well-being, measured through the Self-Rating Anxiety Scale (SAS) and the Self-Rating Depression Scale (SDS) scores; and quality of life, evaluated using the Medical Outcomes Study 36-Item Short-Form Health Survey (SF-36). Additionally, patient satisfaction and the incidence of complications were monitored and compared before and after the intervention.

**Results:**

The results indicated that the observation group exhibited superior outcomes compared to the control group following the intervention, as evidenced by reduced NIHSS scores and elevated BI scores. Post-intervention, both groups experienced a decline in the scores for the SAS and the SDS, with a notably greater reduction observed in the observation group (both *p* < 0.05). Furthermore, after the intervention, improvements were noted in all dimensions of the SF-36 for both groups, yet the observation group demonstrated a more pronounced enhancement (*p* < 0.05). Additionally, the observation group reported a lower incidence of complications and higher levels of nursing satisfaction.

**Conclusion:**

High-quality nursing for patients recovering from hypertensive ICH post-surgery has been shown to have an indirect influence on neurological recovery by alleviating adverse psychological conditions and elevating overall quality of life. This approach is associated with a minimal rate of complications and a high level of nursing satisfaction.

## 1 Introduction

Stroke poses a major threat to China’s public health and is a severe chronic non-communicable disease, characterized by high morbidity, disability, mortality, recurrence, and economic burden (1, 2). Globally, intracranial hemorrhage (ICH)—a key subtype of hemorrhagic stroke—accounts for 8–15% of acute cerebrovascular emergencies requiring urgent surgical evaluation**, highlighting its significance in surgical emergency settings (3). As a prevalent cerebrovascular emergency, ICH has hypertension as its dominant etiological factor (50–70% of cases) and the most common clinical subtype (4, 5). In 2021, the age-standardized incidence rates of ICH varied significantly across different countries, spanning a range from 9.9 to 198.1 cases per 100,000 individuals. Notably, the highest incidence rates were recorded in the Solomon Islands at 198.1 per 100,000, followed by Mongolia with 153.3 per 100,000, and Kiribati at 140.6 per 100,000. Conversely, the lowest incidence rates were found in Switzerland, standing at 9.9 per 100,000, New Zealand at 10.1 per 100,000, and Australia at 10.9 per 100,000 (6). Hypertensive ICH mainly affects middle-aged and elderly people (peak 55–75 years), with high morbidity and mortality due to rapid progression. Hemorrhage location, severity, and lesion extent directly shape clinical manifestations (7). Surgical intervention is the primary management strategy, as it removes intracranial hematomas, eases symptoms, and reduces mortality (8). However, insufficient postoperative care can cause complications like pulmonary infections, hindering neurological recovery. Thus, identifying reliable nursing interventions is critical for ICH patients.

High-quality nursing is an evidence-based, patient-centered care model that delivers exceptional nursing experiences by integrating two core components: (a) standardized physical care (including postoperative vital sign monitoring, professional wound management, and targeted complication prevention) and (b) individualized psychological support (including anxiety/depression alleviation, disease-specific health education, and continuous emotional counseling). This approach emphasizes the compassionate nature of nursing, enhances the overall quality of care patients receive, and ensures holistic, comprehensive services by addressing both physical and emotional needs (9, 10). This specific nursing framework has been extensively utilized in a range of specialized fields, such as Neurology and Cardiology, and has produced positive results. Research by Xiao et al. has demonstrated that high-quality nursing can effectively alleviate negative emotions in patients and enhance their quality of life, while also reducing the incidence of complications (11). Studies conducted by Li et al. elucidate that High-quality nursing intervention has proven to be highly effective for nasopharyngeal carcinoma patients undergoing radiotherapy. It can significantly enhance therapeutic outcomes, reduce the incidence of adverse reactions, and boost patients’ awareness of health-related knowledge. Additionally, it helps alleviate negative emotions and improve patients’ quality of life, sleep quality, and nursing satisfaction (12). Additionally, combining high-quality nursing with respiratory training can significantly enhance pulmonary function, treatment effectiveness, and quality of life in patients with chronic obstructive pulmonary disease (13). Moreover, research conducted by Zuo et al. has demonstrated that high-quality nursing intervention yields substantial benefits in the treatment of elderly cataract patients. These benefits include effectively reducing intraocular pressure, enhancing patients’ quality of life, decreasing the incidence of postoperative complications, and boosting patient satisfaction (14).

However, current literature on high - quality nursing has several limitations. Firstly, most existing studies concentrate on chronic conditions like chronic hypertension disease or malignant tumors such as nasopharyngeal carcinoma. There is a scarcity of research specifically targeting acute cerebrovascular diseases, especially postoperative ICH patients. ICH patients face unique recovery challenges, including rapid neurological function fluctuations and a high risk of early complications in the postoperative period. For instance, a study by Huang et al. (15) showed that ICH patients have a high rate of early - stage deterioration, which requires immediate and specialized nursing care that differs from the long - term, stable - condition management in chronic diseases. Secondly, current research seldom delves into the disease - specific benefits of high - quality nursing. There exists a paucity of empirical research concerning the applicability of high - quality nursing within the context of the postoperative patients diagnosed with ICH. Consequently, the current investigation aims to apply high - quality nursing interventions for this specific population to examine its effects on mental health, neurological functioning, and daily living capabilities. This approach seeks to validate the practicality of this model as a potential nursing intervention program.

## 2 Materials and methods

### 2.1 Subjects

#### 2.1.1 Data collection and assessment

**Figure 1:**
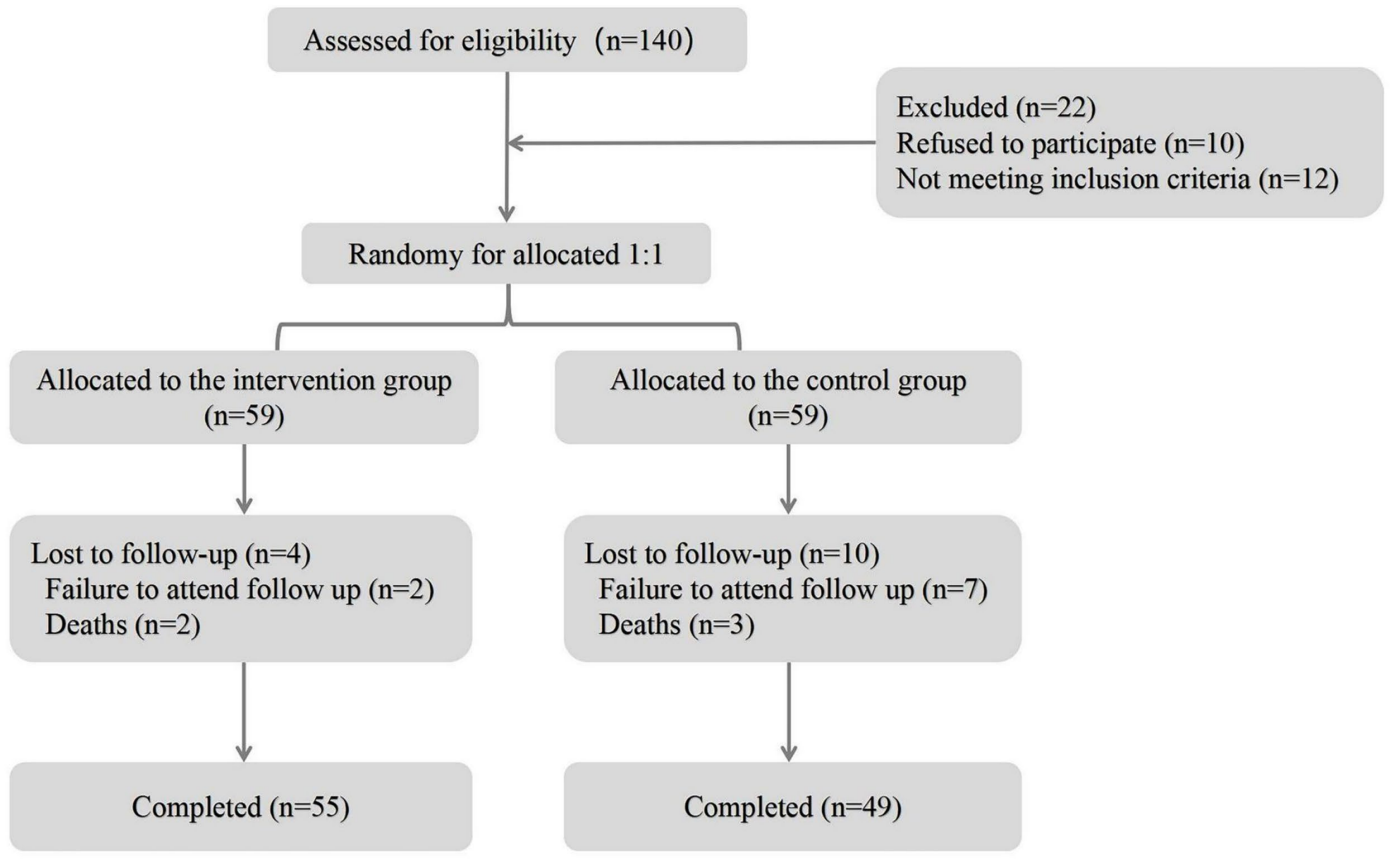
Flowchart of participant selection.

A total of 104 patients with ICH were consecutively recruited from Lixin County Hospital of Traditional Chinese Medicine from August 2019 to January 2025 for the present prospective observational analysis (Figure 1). The sample size was calculated based on the following considerations. We estimated the effect size by referring to previous similar studies on the outcomes of stroke exercise nursing (16). Based on this study, we assumed an expected effect size. Regarding the power level, we set a power of 0.8 (i.e., 80% power), which is a commonly used value in medical research. This means we aimed to have an 80% chance of correctly detecting a true difference if it exists. With the assumed effect size and the set power level, along with a significance level of 0.05 (a standard choice for two-tailed tests), we used a formula for sample size calculation suitable for comparing two independent groups. In the present study, participants were randomly allocated to either the control group or the intervention group using a computer-generated randomization sequence with a block size of 4 to ensure balance between the groups. The randomization sequence was generated by an independent statistician who was not involved in the recruitment or treatment of participants. Allocation concealment was implemented using sequentially numbered, opaque, sealed envelopes. Once a participant was deemed eligible and had provided informed consent, the next envelope in the sequence was opened to reveal the group assignment. Regarding blinding, the participants were not blinded as they were aware of the treatment they received (either standard care in the control group or the additional intervention in the intervention group). However, the outcome assessors were blinded. The assessors who evaluated the patients’ outcomes (such as functional status, complication occurrence) were not informed of the group assignment of the patients they were assessing. This was done to reduce potential bias in outcome evaluation. We followed the CONSORT (Consolidated Standards of Reporting Trials) guidelines which emphasize the importance of blinding in reducing bias in randomized controlled trials, and although our study was a prospective observational analysis with random allocation, we applied the relevant blinding principles from these guidelines (17). The study protocol received ethical approval from the Institutional Review Board of Lixin County Hospital of Traditional Chinese Medicine (Approval No. LXCM-IRB-2019-012; Date: 15 July 2019), with written informed consent obtained from all conscious participants or their legally authorized representatives using standardized proxy consent forms. This process strictly adhered to the ethical principles outlined in the Declaration of Helsinki. Exclusion criteria were strictly applied as follows: (1) patients manifesting profound consciousness impairment (Glasgow Coma Scale score < 9); (2) coexistence of critical comorbidities including active systemic infections, end-stage hepatic/renal insufficiency, immune-mediated disorders, hematologic malignancies, rheumatologic conditions, or metastatic neoplasms; (3) cases requiring premature termination of surgery due to uncontrollable intraoperative hemorrhage or vital sign instability. Participants under consideration met the following criteria: (1) age greater than 18 years and less than 80 years; (2) patients who meet the clinical diagnostic criteria for ICH and have been confirmed by imaging (18); (3) All patients were eligible for surgical treatment.

### 2.2 Nursing methods

The control group received conventional nursing encompassing clinical status assessments, medication management, dietary and activity recommendations, psychosocial support, health education. The observation group implemented high-quality nursing protocol alongside conventional nursing, which involved integrating evidence-based interventions into the nursing workflow to optimize key postoperative care processes for hypertensive ICH patients. The senior responsible nurses developed a standardized care plan and provided targeted training sessions across all shifts, ensuring all nurses achieved proficiency in specialized clinical procedures.

#### 2.2.1 Establishment of high-quality nursing team

The leader of the quality control team held the position of a national manager for brain and heart health, while the team consisted of municipal managers specializing in brain and heart health, neurocritical care nurses, nurses certified in stroke care, rehabilitation therapists, and psychological specialist nurses. The members of the clustering care team were duly qualified to oversee and provide guidance in the care of patients within the observation group for the duration of the entire process.

#### 2.2.2 Admission health education

Upon admission, family members were comprehensively educated on the etiology of ICH, surgical interventions, postoperative recovery guidelines, and critical nursing strategies, with illustrated educational materials provided for reference. During the post-awakening phase, multimodal health education (e.g., video demonstrations and visual infographics) was delivered to enhance patient comprehension and adherence.

#### 2.2.3 Basic nursing

The patient was repositioned at 2-h intervals with supportive soft pillows placed under high-risk pressure areas. Postoperative nutritional management followed a phased protocol, transitioning from liquid to solid diet under clinical supervision. Concurrently, routine oral hygiene care was performed to clear secretions and maintain mucosal integrity. Lower extremity monitoring included daily tracking of skin temperature, color, tissue turgor, and bilateral circumference measurements. Patients were instructed on evidence-based compression therapy (elastic stockings) and guided self-lymphatic drainage techniques. Surgical site dressings were changed aseptically per schedule, with systematic evaluation for erythema, edema, or exudate.

#### 2.2.4 Psychological nursing

ICH represents a neurosurgical emergency requiring immediate intervention, with postoperative recovery often complicated by concurrent speech and motor dysfunction coupled with significant psychopathological manifestations including anxiety disorders and illness-related phobias. This clinical profile necessitates the implementation of specialized psychiatric nursing protocols to address multidimensional patient needs. The nursing team conducted psychological counseling sessions for patients in the observation group each morning and evening. They encouraged patients experiencing unsatisfactory treatment outcomes to maintain their confidence and collaboratively address the challenges encountered during rehabilitation. This supportive approach aimed to foster active patient participation in their treatment, ultimately leading to improved outcomes.

#### 2.2.5 Functional exercise

The nursing team should provide comprehensive care for patients with varying risk levels, including intermediate-risk, high-risk, and ultra-high-risk patients. In cases where the patient’s condition is stabilized, it is generally recommended to initiate rehabilitation training at an early stage, typically within 48 h to 2 weeks post-stabilization. Early guidance should be provided to the patient’s family regarding the implementation of passive exercises until the patient is able to get out of bed and ambulate independently. Subsequently, the family should be instructed on facilitating activities for the affected side of the upper limb, including elbow flexion, fist clenching, and stretching. Similar guidance should be extended to the affected lower limb, focusing on passive movements of the ankle, knee, and hip joints. Additionally, it is recommended to perform muscle massage on the lower limbs from distal to proximal, twice daily for 15 min each session. The rehabilitation therapist will conduct a comprehensive evaluation of the patient’s condition and will gradually and systematically increase the exercise intensity. In addition, active ankle pump exercises can be performed to help patients relax their legs. These exercises should be done slowly, allowing patients to experience mild discomfort. Patients should aim to step down on their toes as much as possible, holding this position for 5 s before returning to a neutral position. Subsequently, they should flex their toes upward and perform foot rotary exercises. While maintaining extended legs, the ankle joint should serve as the pivot point for rotations in both clockwise and counterclockwise directions. This routine should be practiced 2–3 times per day, with each session consisting of 20–50 repetitions. In addition, the use of rehabilitation training equipment to assist exercise, through the pressure cycle therapy instrument until the affected side can move, from the bottom to the top, the hemiplegic side of the torso sequentially inflatable pressure, 2 times a day, each time 30 min, 2 weeks for a course of treatment; lower limb power car, relax the hip, knee, and ankle joints, contraction of the lower limb muscles, 2 times a day, each time 20 min, and in accordance with the degree of tolerance, and gradually increase the speed of the exercise.

#### 2.2.6 Nursing of complication

1. Pulmonary infection: Individuals experiencing ICH often encounter limitations in respiratory function due to dysphagia and prolonged periods of immobility. As a result, respiratory secretions may accumulate in the pulmonary system for extended durations, promoting the proliferation of pathogenic bacteria in the lungs and increasing the likelihood of aspiration episodes. This situation markedly elevates the risk of developing pulmonary infections. In the absence of contraindications, it is recommended that the patient be positioned in a semi-recumbent posture, with the upper torso elevated at an angle between 30° and 40°. Timely turning and gentle tapping on the back should be performed to facilitate sputum expulsion. Additionally, nebulization therapy should be administered as necessary. It is crucial to assess the patient’s swallowing capabilities using the water swallow test; if the results indicate a score of grade III or higher, the insertion of a gastric tube should be performed according to the physician’s directives to mitigate the risk of choking and aspiration. Furthermore, routine oral hygiene should be conducted every morning and evening.
2. Stress ulcers: Carefully monitor the patient’s condition and any potential complications. Pay particular attention to the insertion of the gastric tube, and assess whether there is any bloody or coffee-colored fluid being aspirated. Additionally, observe for signs of hemorrhagic shock and conduct a fecal occult blood test. If any abnormalities are detected, implement emergency measures promptly.
3. Pressure ulcer: Prolonged bed rest can lead to localized tissue ischemia and hypoxia, resulting in the formation of pressure ulcers, which can hinder patient recovery. Therefore, it is essential for patients to be repositioned regularly, ideally every 2 h, to mitigate these risks. Additional measures include patting the back, performing massages, maintaining clean and dry bed linens, and utilizing air mattresses as early as possible to prevent the occurrence of pressure ulcers.
4. Lower extremity venous thrombosis: Postoperatively, patients should be closely monitored for changes in vital signs. Risk factor assessments for deep vein thrombosis (DVT) in both lower limbs should be conducted biweekly. Additionally, coagulation function and D-dimer levels must be regularly monitored in patients categorized as intermediate-risk, high-risk, and ultra-high-risk. The circumference of both lower limbs should also be measured twice a week. Carefully monitor the patient for symptoms such as chest tightness, chest and back pain, loss of consciousness, and hemoptysis, as these may indicate pulmonary embolism. If necessary, conduct a pulmonary arteriography CT examination. Additionally, closely observe the patient’s lower limb skin color and the pulsation of the dorsalis pedis artery, checking for swelling and pain to remain vigilant for the occurrence of lower limb deep vein thrombosis. If indicated, perform a lower limb ultrasound examination to facilitate early detection and timely treatment.

#### 2.2.7 Continuing nursing

Following patient discharge from the hospital, nursing staff implemented a comprehensive follow-up strategy that included online guidance, weekly telephone check-ins, and in-person visits at three-month intervals. This approach aimed to oversee and direct patients’ adherence to medication regimens, dietary choices, and rehabilitation efforts. The objective was to ensure that patients adhered to their prescribed medication schedules, engaged in proactive recovery practices, avoided detrimental lifestyle habits, and received prompt assistance for any emerging issues.

### 2.3 Clinical data collection

Baseline characteristics include demographic data (including age, gender, education years, married), clinical data (including the history of hypertension, diabetes mellitus, smoking, and drinking) were collected for patients at admission. Venous blood was drawn from patients the next morning after admission. We measured white blood cell (WBC) counts, glucose (G), triglyceride (TC), triglycerides (TG), high-density lipoprotein (HDL), low-density lipoprotein (LDL), apolipoprotein A (ApoA), and apolipoprotein B (ApoB).

### 2.4 Observation indicators

Patients were asked to return to the hospital for a follow-up appointment 3 months post-intervention. The pertinent evaluation scales were administered to the patients prior to the intervention (upon admission) and subsequently 3 months post-onset. In instances where patients were unable to attend follow-up appointments as scheduled, nursing staff facilitated the completion of the forms via follow-up telephone calls.

1. Psychological parameters: the assessment of anxiety and depression in patients was conducted utilizing the Self-rating Anxiety Scale (SAS) and the Self-rating Depression Scale (SDS) (19). As the scores ascended, there was a corresponding rise in the levels of anxiety and depression. The two assessment tools comprised 20 items each, evaluated on a four-point scale that spanned from 0 to 100, where lower scores were indicative of a more favorable psychological condition.
2. Neurological functionality: the National Institutes of Health Stroke Scale was used to assess the neurological function of the patients in both groups on the day of admission and at 3 months of intervention, with a total score of 0 to 35, with higher scores suggesting that the patients had more severe neurological deficits (20).
3. Activities of Daily Living (ADL): The Barthel scores was used to evaluate the daily life ability of the two groups of patients, which consists of 10 items, with a total score of 0–100, the higher the score, the better the patient’s daily life ability (21).
4. Quality of Life Metrics: The Medical Outcomes Study 36-item short-form health survey (SF-36) was administered pre- and post-intervention, facilitating a comparative analysis between the control and experimental cohorts (22).
5. Patient Satisfaction: A customized “Patient Clinical Care Satisfaction Questionnaire” was employed to evaluate the degree of patient satisfaction following the intervention in both groups. This assessment instrument consists of five items, allowing for a total score that can reach 100. A higher score indicates increased satisfaction regarding the nursing care provided. Furthermore, occurrences of clinical complications following the intervention were carefully recorded and analyzed comparatively between the groups.

### 2.5 Statistical analysis

Statistical investigation was carried out using SPSS for Windows (version 26.0, Inc., Chicago, IL, USA). Continuous variables that follow a normal distribution are characterized by their means and standard deviations. In contrast, continuous variables exhibiting a non-normal distribution are typically described using median values and the interquartile range (IQR). Categorical variables, on the other hand, are represented by percentages and frequencies. The Kolmogorov–Smirnov test was conducted to assess the normality of the distributions. Continuous variables exhibiting a normal distribution were analyzed through the t-test, whereas continuous variables that did not conform to a normal distribution were assessed using the Mann–Whitney *U*-test. Additionally, categorical variables were compared across groups employing either the Fisher’s exact test or the Pearson chi-squared test as appropriate. The distinctions between the two groups regarding the evaluation of abnormal distributions of continuous variables were evaluated using the Mann–Whitney *U* test. A *p*-value of less than 0.05 was considered statistically significant.

## 3 Results

### 3.1 Clinical and demographic characteristics of patients in observation group and control group

A cohort of 104 patients participated in this investigation, comprising 49 individuals in the control group (27 males) and 55 individuals in the observation group (30 females). The average age for the control group was recorded as (68.41 ± 7.22) years, while the observation group had a mean age of (67.45 ± 7.24) years, indicating no statistically significant difference between the two groups (*p* > 0.05). Additionally, other baseline characteristics did not reveal any notable differences between the participant groups, with the findings detailed in Table 1.

**Table 1:** Clinical and demographic characteristics of patients in observation group and control group.

Variables	Observation group (*n* = 55)	Control group (*n* = 49)	*P-*value
Demographic characteristics			
Gender, Males, *n* (%)	30 (54.54)	27 (55.10)	0.955
Age, years, mean ± SD	67.45 ± 7.24	68.41 ± 7.22	0.503
Married, *n* (%)	45 (81.82)	44 (89.79)	0.248
Vascular risk factors (%)			
Hypertension	40 (72.73)	40 (81.63)	0.282
Diabetes mellitus	20 (36.36)	22 (44.89)	0.376
Coronary heart disease	10 (18.18)	7 (14.29)	0.592
Atrial fibrillation	11 (20)	13 (26.53)	0.430
current smoking	40 (72.73)	38 (77.55)	0.571
Alcohol consumption	24 (43.64)	28 (57.14)	0.169
Laboratory findings (IQR)			
WBC, ×10^9^/L, mean ± SD	7.66 ± 1.41	7.71 ± 1.66	0.879
Glucose, mmol/L, mean ± SD	6.15 ± 2.88	6.75 ± 2.66	0.274
TG, mmol/L, mean ± SD	1.46 ± 0.57	1.44 ± 0.68	0.879
TC, mmol/L, mean ± SD	4.47 ± 0.96	4.89 ± 1.38	0.073
HDL-C, mmol/L, mean ± SD	1.05 ± 0.29	1.17 ± 0.35	0.059
LDL-C, mmol/L, mean ± SD	2.61 ± 0.76	2.72 ± 0.79	0.468
ApoA, g/L, mean ± SD	1.36 ± 0.29	1.27 ± 0.22	0.072
ApoB, g/L, mean ± SD	0.90 ± 0.25	1.01 ± 0.88	0.372
Hematoma location, *n* (%)			
Frontal lobe	4 (7.27)	3 (6.12)	0.815
Parietal lobe	3 (5.45)	3 (6.12)	0.884
Temporal lobe	5 (9.09)	5 (10.20)	0.848
Occipital lobe	4 (7.27)	4 (8.16)	0.865
Basal ganglia	30 (54.55)	26 (53.06)	0.880
Brainstem	3 (5.45)	4 (8.16)	0.582
Cerebellum	6 (10.91)	4 (8.16)	0.635

WBC, white blood cell, G, glucose, TC, triglyceride, TG, triglycerides; HDL, high-density lipoprotein; LDL, low-density lipoprotein; ApoA, apolipoprotein A; ApoB, apolipoprotein B.

### 3.2 The SAS and SDS scores between the two groups of patients

Negative emotions were assessed in both groups prior to and following the intervention utilizing the SAS and SDS scales. The initial evaluation indicated no significant difference in SAS and SDS scores between the two groups (all *p* > 0.05, *t* = 0.44 and *t* = 0.32). Post-intervention results demonstrated a significant enhancement in SAS and SDS scores for both groups; however, the degree of improvement was notably greater in the observation group (all *p* < 0.05) (Figure 1).

### 3.3 Comparison of SF-36 scores between the two groups

Prior to the intervention, no significant differences were observed in the scores across the eight domains of physical functioning, including role physical, bodily pain, general health, vitality, social functioning, role emotional, and mental health, between the observation group and the control group (all *p* > 0.05). Following the intervention, there was a noticeable enhancement in the scores for all dimensions of the SF-36 in both groups; however, the observation group exhibited higher scores across all measures (all *p* < 0.05; Table 2).

**Table 2:** Comparison of SF-36 scores before and after intervention.

Time	Group	Physical function	Role physical	Bodily pain	General health	Vitality	Social functioning	Role emotional	Mental health
Before intervention	Observation group (*n* = 55)	55.75 ± 3.65	58.73 ± 4.45	60.47 ± 5.20	54.64 ± 5.33	54.87 ± 5.42	60.47 ± 5.24	55.51 ± 4.66	58.87 ± 5.74
	Control group (*n* = 49)	54.84 ± 3.83	58.14 ± 4.65	59.47 ± 4.58	52.76 ± 5.33	55.10 ± 4.14	61.78 ± 4.50	54.90 ± 4.58	58.57 ± 7.30
	*t*-value	−1.238	−0.655	−1.038	−1.795	0.240	1.350	−0.674	−0.235
	*P-*value	0.218	0.514	0.302	0.076	0.811	0.180	0.502	0.815
	95% CI	−1.83 to 1.15	−2.38 to 1.11	−1.42 to 2.77	−0.17 to 4.01	−2.52 to 0.61	−2.25 to 1.38	−2.25 to 1.41	−2.55 to 2.73
After intervention	Observation group (*n* = 55)	81.18 ± 5.76	77.88 ± 4.91	79.36 ± 5.71	78.85 ± 6.53	81.35 ± 7.29	81.84 ± 6.56	80.87 ± 6.51	78.49 ± 5.51
	Control group (*n* = 49)	77.27 ± 6.18	75.20 ± 3.62	76.31 ± 5.84	75.41 ± 6.80	78.22 ± 4.89	78.31 ± 4.55	76.12 ± 7.38	76.06 ± 5.01
	*t*-value	−3.345	−3.133	−2.695	−2.633	−2.533	−3.152	−3.488	−2.344
	*P-*value	0.002	0.002	0.008	0.010	0.013	0.002	0.001	0.021
	95% CI	1.56 to 6.72	0.57 to 4.09	0.67 to 5.41	0.25 to 4.50	0.42 to 4.95	1.19 to 5.56	1.74 to 7.76	0.76 to 4.70

### 3.4 Comparison of NIHISS and Barthel index scores between the two groups

The findings presented in Table 3 indicate that there was no statistically significant difference in the NIHSS and BI scores between the two patient groups at the time of admission (all *p* > 0.05). However, post-intervention analysis demonstrated that the NIHSS scores for the observation group were significantly lower than those of the control group, while the Barthel index scores for the observation group were significantly higher than those of the control group (all *p* < 0.05).

**Table 3:** Comparison of NIHSS scores and Barthel index score between the two groups.

Group	NIHSS scores		Barthel index score	
	At the time of admission	After intervention	At the time of admission	After intervention
Observation group	10.31 ± 2.48	5.64 ± 1.99	33.64 ± 6.62	70.09 ± 10.96
Control group	10.39 ± 3.22	7.29 ± 3.03	32.84 ± 5.19	56.82 ± 15.28
*t*-value	0.140	3.319	−0.314	−5.131
*P* value	0.889	0.001	0.754	0.000
95% CI	−0.48 to 1.63	−3.24 to 1.15	−3.66 to 5.18	8.44 to 18.73

### 3.5 Comparison of complication rates between the two groups

At the 3-month follow-up assessment, the observation group reported two significant complications, both of which were instances of pulmonary infection. In comparison, the control group experienced a total of five pulmonary infections, as well as one case of stress ulcers, one instance of pressure ulcers, one case of lower extremity venous thrombosis, and one reported death. The findings indicated that the overall rate of complications in the observation group was significantly lower than that observed in the control group (*p* < 0.05, Table 4).

**Table 4:** Comparison of complication rates between the two groups.

Group	Observation group (*n* = 55)	Control group (*n* = 49)	χ^2^ value	*P-*value
Pulmonary infection	2 (3.64)	5 (10.20)	–	–
Stress ulcers	0 (0)	1 (2.04)	–	–
Pressure ulcer	0 (0)	1 (2.04)	–	–
Lower extremity venous thrombosis	0 (0)	1 (2.04)	–	–
Death	0 (0)	1 (2.04)	–	–
Total incidence	2 (3.64)	9 (18.37)	5.945	0.015

### 3.6 Comparison of nursing satisfaction between the two groups

**Figure 2:**
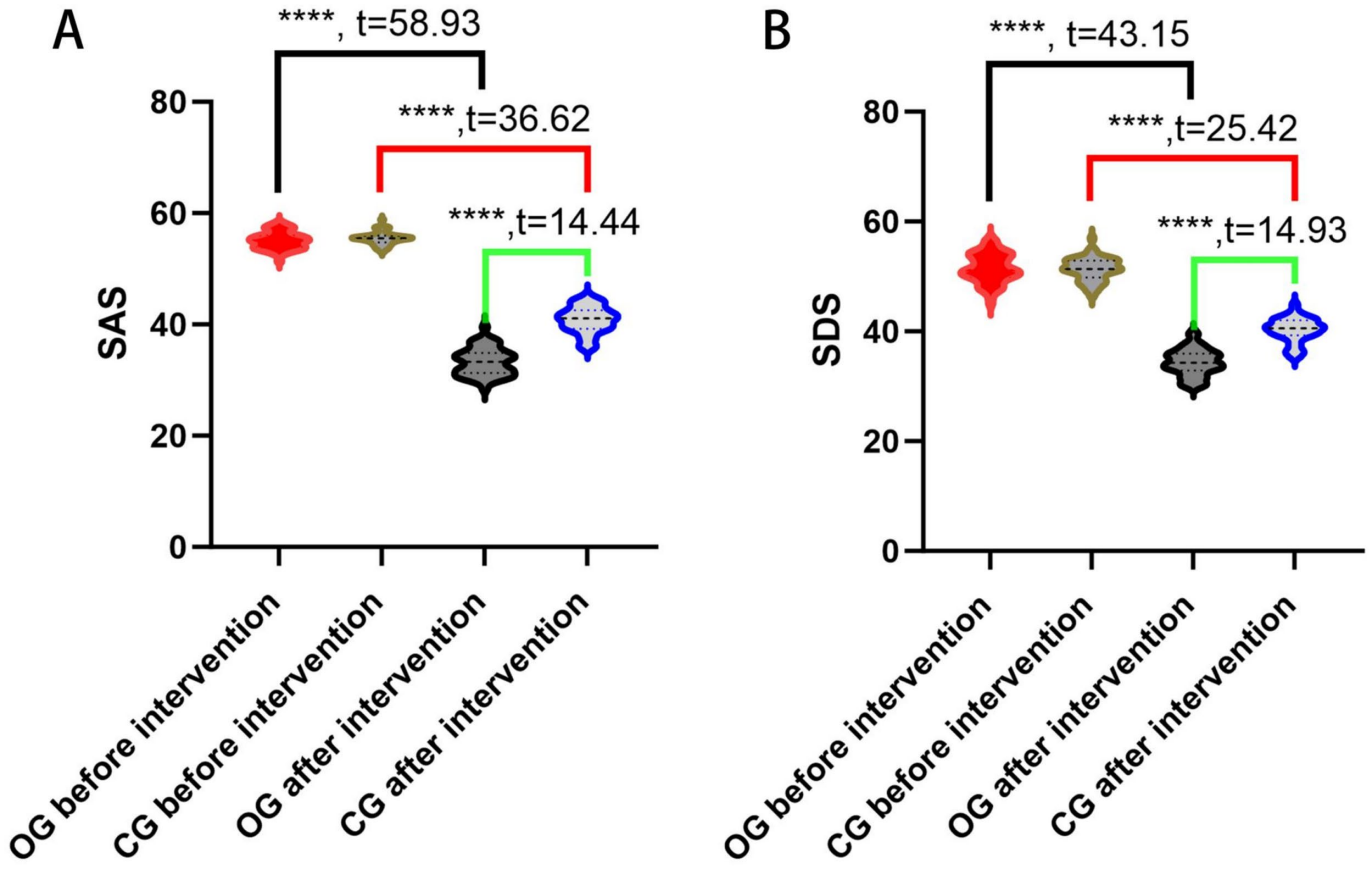
Comparison of the SAS and SDS scores. **(A)** SAS scores of the two groups before and after intervention; **(B)** SDS scores of the two groups before and after intervention. OG, observation group; CG, control group; SAS, self-rating anxiety scale; SDS, self-rating depression scale; ****indicates *p* < 0.0001.

In the observation group, a total of 35 patients expressed a high level of satisfaction with the nursing care they received, with 19 indicating satisfaction and only 1 reporting dissatisfaction. This resulted in an impressive nursing satisfaction rate of 98.18%. Conversely, in the control group, 24 patients reported being very satisfied with the nursing care, while 18 were satisfied and 7 expressed dissatisfaction, leading to a nursing satisfaction rate of 85.71%. Notably, the nursing satisfaction rate among patients in the observation group was significantly higher than that in the control group. Statistical analysis confirmed that this difference was significant (all *p* < 0.05, Table 5 and Figure 2).

**Table 5:** Comparison of the nursing satisfaction [*n*(%)].

Group	Observation group (*n* = 55)	Control group (n = 49)	*χ*2 value	*P-*value
Very satisfied	35 (63.64)	24 (48.98)	–	–
Satisfied	19 (34.55)	18 (36.73)	–	–
Dissatisfied	1 (1.82)	7 (14.28)	–	–
Nursing satisfaction	54 (98.18)	42 (85.71)	5.673	0.017

## 4 Discussion

To the best of our knowledge, this is the first study to explore the application of high-quality nursing for patients with postoperative ICH. Hypertensive ICH, a prevalent clinical emergency, is characterized by high disability and mortality rates, which exert a direct adverse impact on patients’ quality of life and family well-being. A notable demographic feature of this patient population is the predominance of elderly individuals; as they age, their physical functions gradually decline, leading to reduced responsiveness to conventional care. This unique combination of disease severity and demographic vulnerability has made the optimization of clinical nursing for elderly hypertensive ICH patients a critical priority in current clinical practice.

Simultaneously, China’s rapid economic development has driven a significant shift in public health demands. Beyond effective disease treatment, patients increasingly prioritize a comfortable and patient-centered experience throughout the treatment journey. Recognizing nursing services as a core component of the patient care continuum, leading medical institutions in China have proactively initiated reforms in nursing management systems. These reforms aim to streamline service processes, enhance nursing quality, and improve service efficiency, aligning with the evolving expectations of patients and the broader goals of healthcare system advancement.

Negative psychological emotions, including anxiety, depression, tension, and emotional distress, typically arise when individuals perceive a discrepancy between expected and actual outcomes of events or interpersonal interactions (23, 24). For ICH patients, such psychological sequelae are particularly prevalent: intracranial hemorrhage is one of the most disabling and life-threatening neurological disorders, and post-stroke depression (PSD) affects approximately 15–23% of ICH survivors (25, 26). Multiple factors contribute to the development of negative emotions in this population: concerns about the substantial economic burden of long-term treatment, anxiety regarding the disease’s impact on personal independence, and guilt about the caregiving burden imposed on family members (27). The consequences of unresolved psychological issues are far-reaching—patients with PSD exhibit higher risks of cognitive impairment, stroke recurrence, and increased mortality (28–30).

In our study, after the implementation of high-quality nursing intervention, the SAS and SDS scores of the observation group were significantly lower than those of the control group. This finding confirms that high-quality nursing can effectively alleviate negative psychological states in ICH patients. The underlying mechanism may be attributed to the patient-centered philosophy of high-quality nursing: it incorporates empathetic communication and therapeutic relationship-building to strengthen the patient–nurse alliance, creating a safe space for patients to express emotional distress. Moreover, dedicated nurses, through continuous observation and interaction, gain insight into patients’ individual concerns (e.g., fear of disability, financial worries) and provide timely, personalized psychological support tailored to these specific needs. This result is consistent with existing literature: Wang et al. demonstrated that high-quality nursing reduced anxiety, depression scores, and psychological distress in postoperative patients with advanced non-small cell lung cancer (31); a systematic review also provided evidence that high-quality nursing alleviated depression and anxiety in patients with thyroid cancer during the perioperative period (32), and another meta-analysis summarized similar positive effects of high-quality nursing on psychological disorders in patients with ovarian cancer during the perioperative period (33). Notably, our study extends this body of evidence by validating the effectiveness of high-quality nursing in the unique context of ICH, a population with distinct psychological stressors related to neurological disability and functional decline.

Neurological function recovery and functional independence are key clinical outcomes for ICH patients. In our study, the NIHSS score of the observation group after intervention was significantly lower than that of the control group, while the BI score was significantly higher. This indicates that high-quality nursing substantially improves neurological function and functional independence in ICH patients. The probable reasons for this improvement are multi-faceted: first, high-quality nursing integrates multiple evidence-based care measures, developed based on a comprehensive assessment of each patient’s disease severity, comorbidities, and functional status. Second, for specific clinical problems (e.g., risk of pressure ulcers, delayed motor recovery), the nursing team develops joint care plans supported by the latest clinical evidence, ensuring targeted and effective interventions. Third, high-quality nursing includes systematic health education, which enhances patients’ understanding of hypertensive ICH—covering disease pathophysiology, surgical procedures (if applicable), treatment goals, and self-care requirement—empowering patients to actively participate in their recovery. Additionally, practical interventions such as positional care and regular massage improve blood circulation in pressure-sensitive areas, reducing the risk of pressure ulcers and associated complications; early functional exercises, tailored to each patient’s neurological status, promote the recovery of impaired nerve function and prevent muscle atrophy.

Our findings align with and expand upon previous research: Huang et al. (34) found that combining low-frequency pulsed electrical stimulation with early whole-body functional exercise (guided by high-quality nursing) improved limb function in patients with brachial plexus injuries; Narigele et al. (35) discovered that integrating the Montessori technique with high-quality nursing accelerated joint function recovery and improved quality of life in patients with clavicle fractures. Unlike these studies, which focused on musculoskeletal or peripheral nerve disorders, our research demonstrates that a multi-component high-quality nursing approach is equally effective in promoting neurological recovery in ICH patients—a population with more complex and severe neurological impairments.

Long-term recovery and medication adherence are persistent challenges in ICH management. Patients with ICH require an extended recovery period and long-term oral medication (e.g., antihypertensives to prevent recurrence), but suboptimal medication adherence is common, often due to insufficient disease knowledge, forgetfulness, or concerns about medication side effects. Our study found that patients in the high-quality nursing group had significantly higher scores in all dimensions of the SF-36 (*p* < 0.05) compared to the control group. This suggests that high-quality nursing not only enhances the overall quality of life of patients with hypertensive ICH but also improves their medication adherence. The improvement in adherence may be attributed to the continuous follow-up and personalized education components of high-quality nursing: nurses regularly monitor medication intake, address patients’ concerns about side effects, and use reminder tools (e.g., medication calendars) to support adherence. This result is consistent with a study by Feng et al., which showed that combining high-quality nursing with health education in chemotherapy regimens for non-small cell lung cancer improved patient compliance, self-management ability, and physical and psychological health (36).

The novelty of this study lies in two key aspects: first, it is the first study to systematically evaluate the application of high-quality nursing in patients with hypertensive ICH, filling a gap in the existing literature that primarily focuses on high-quality nursing in oncology or musculoskeletal populations. Second, unlike previous studies that only assessed single outcomes (e.g., psychological status or functional recovery), our research adopts a comprehensive outcome framework, evaluating the impact of high-quality nursing on neurological function (NIHSS), functional independence (Barthel Index), psychological well-being (SAS, SDS), quality of life (SF-36), complication rates, and patient satisfaction. This comprehensive assessment provides a holistic understanding of the intervention’s effectiveness.

Based on the study findings, we propose the following recommendations for clinical management and nursing education: (1) Clinical Management: high-quality nursing should be integrated into the standard care protocol for patients with hypertensive ICH. Medical institutions can establish specialized high-quality nursing teams consisting of nurses with expertise in neurology, psychological counseling, and rehabilitation. These teams should implement the multi-component intervention (comprehensive assessment, evidence-based care plans, psychological support, functional exercises, and medication adherence monitoring) throughout the patient’s hospital stay and extend it to post-discharge follow-up (e.g., via telephone or home visits) to maintain long-term effectiveness. (2) Nursing Education: Nursing education programs should incorporate training on high-quality nursing for neurological disorders, with a specific focus on ICH. Training content should include: (a) comprehensive assessment skills for ICH patients (e.g., neurological function evaluation, psychological state screening); (b) evidence-based interventions (e.g., early functional exercise protocols, personalized psychological support techniques); and (c) strategies to improve medication adherence (e.g., patient education, reminder tools). Additionally, continuing education courses for practicing nurses should be developed to update their knowledge on high-quality nursing and ensure the consistent implementation of high-quality care.

Despite its contributions, this study has several limitations. First, it was a single-center study with a relatively small sample size, which may limit the generalizability of the findings and introduce regional biases (e.g., variations in patient demographics or healthcare practices across different regions of China). Future studies should adopt a multicenter design with a larger sample size to validate our results. Second, the high-quality nursing intervention was only implemented for 3 months, and long-term follow-up (e.g., 6 or 12 months) was not conducted. This prevents us from evaluating the long-term effects of the intervention on outcomes such as stroke recurrence, long-term medication adherence, and quality of life. Future research should include extended follow-up periods to address this gap.

## 5 Conclusion

In conclusion, the high-quality nursing model in this study—comprising personalized psychological support, tailored neurological rehabilitation, systematic disease education, and continuous complication monitoring—shows potential for improving outcomes in patients with ICH. Based on this study’s findings, the model may mitigate adverse psychological states, facilitate neurological recovery, elevate quality of life, increase nursing satisfaction, and help reduce complication incidence. Given these preliminary results, the model merits consideration for further evaluation and potential promotion in clinical ICH care.

## Data Availability

The original contributions presented in the study are included in the article/supplementary material, further inquiries can be directed to the corresponding author/s.
